# Two Decades of Ureteric Calculus Burden in Australia: National Trends in Incidence, Management, and Demographic Shifts

**DOI:** 10.7759/cureus.103609

**Published:** 2026-02-14

**Authors:** Aqeel Roomy, Taha Mollah, Martin Onotevu, Ahmed Nada, Mohammad Habbal, Badr Rafi, Frans Pretorius

**Affiliations:** 1 General Surgery, Goulburn Valley Health, Shepparton, AUS; 2 General Surgery, Saint Vincent Hospital, Melbourne, AUS; 3 General Surgery, Western Health, Melbourne , AUS; 4 Urology, Ministry of the National Guard – Health Affairs, Jeddah, SAU

**Keywords:** australia, endourology, epidemiology, obstructive hydronephrosis, population trends., public hospital utilisation, stone disease burden, ureteric calculi, ureteroscopy, urolithiasis trends

## Abstract

Objective: To examine national trends in the incidence, management patterns, and demographic characteristics of patients presenting with ureteric calculi in Australia over a 19-year period.

Methodology: A retrospective population-based analysis was performed using three national datasets: the Australian Institute of Health and Welfare (AIHW), the Medicare Benefits Schedule (MBS), and the Australian Bureau of Statistics (ABS). Admissions and procedures associated with ureteric calculi from 2000-2001 to 2018-2019 were identified using the International Classification of Diseases, 10th Revision (ICD-10), and Australian Classification of Health Interventions (ACHI)/MBS procedural codes. Incidence rates were population-adjusted, and temporal trends were assessed using linear regression.

Results: Population-adjusted incidence of ureteric calculi rose by 95% (85.6 to 166.9 per 100,000) over the study period. Procedural interventions increased by 90% (34.0 to 64.7 per 100,000), while the national intervention rate remained stable at approximately 36%. Diagnoses of hydronephrosis with calculus obstruction showed the largest increase (481%). Public hospitals experienced a disproportionate rise in interventions, particularly endoscopic stone destruction (323% increase versus 48% in private hospitals). Interventions increased among adults ≥65 years and women. Same-day discharge rates increased for both conservative and interventional pathways, converging at approximately 45% by 2018-2019.

Conclusions: Australia has experienced a substantial rise in ureteric calculus burden accompanied by proportional increases in procedural interventions. Sector-specific and demographic shifts highlight evolving patterns in stone disease and healthcare utilization, with significant implications for future resource planning.

## Introduction

The global prevalence of urolithiasis has risen sharply in recent decades, with lifetime risk now estimated at up to 12% [1,2]. Lifestyle and metabolic changes, including rising obesity rates, dietary patterns, and reduced physical activity, have contributed to increasing rates of urinary stone disease in both developed and developing countries [2-5]. Parallel increases in emergency presentations, hospital admissions, and stone-related interventions have been consistently reported [6-8].

In light of these global trends, ureteric calculi represent a clinically significant subset of stone disease. As the leading cause of acute renal colic, ureteric stones typically present symptomatically and almost always require hospital-based evaluation [8]. Unlike asymptomatic intrarenal stones, which are inconsistently captured in administrative datasets, ureteric calculus admissions serve as a robust indicator of the symptomatic stone burden at a population level [9].

Although international studies from the United Kingdom [10,11], the United States, and Canada [12,13] have examined long-term trends in ureteric stone management, contemporary Australian literature remains limited. Previous Australian studies have been limited by reliance on private sector data, outdated reporting periods, or lack of population-adjusted analysis [6,9]. Internationally, several reports describe procedural rates rising faster than incidence, driven by improved imaging utilization and expanding endoscopic capability [10-12].

To address the lack of contemporary Australian data, we conducted a comprehensive 19-year national analysis of ureteric calculus admissions and interventions. By evaluating trends in incidence, management, demographics, and sector-specific utilization across publicly available national databases, this study provides the most detailed assessment to date of ureteric calculi in Australia. The findings may inform clinical practice, service planning, and the development of endourological capacity in Australia and comparable health systems.

## Materials and methods

This retrospective population-based study examined national trends in ureteric calculus admissions and interventions in Australia between the 2000-2001 and 2018-2019 financial years. The Study setting comprised all public and private hospitals nationwide. Three publicly available Australian Government datasets were used to capture case numbers and procedural activity: population data from the Australian Bureau of Statistics (ABS) [14], hospital admissions and procedure activity from the Australian Institute of Health and Welfare (AIHW) [15], and privately billed procedural claims from the Medicare Benefits Schedule (MBS) [16]. Together, these datasets capture the vast majority of symptomatic ureteric calculus presentations and interventions performed nationally.

Case identification and coding 

Ureteric calculus admissions were identified using ICD-10 diagnostic codes, while procedural interventions were identified using ACHI (public and private hospitals) and MBS item numbers (private sector). Coding definitions are summarized in Table 1. Differences between databases reflect their respective coding systems. Diagnoses captured symptomatic ureteric obstruction, including hydronephrosis with calculus obstruction. Asymptomatic intrarenal stones, which are inconsistently coded and not reliably captured in administrative datasets, were not included. Procedures were classified as interventional if any endoscopic or open ureteric stone procedure code was recorded. Admissions without recorded procedures were classified as conservative management. Because the AIHW captures all public and private hospital activity and the MBS captures privately billed inpatient procedures, combining these datasets enabled differentiation between public and private sector interventions.

**Table 1:** Coding definitions for ureteric calculus-related diagnoses and procedures. MBS, Medicare Benefits Schedule; AIHW, Australian Institute of Health and Welfare; ACHI, Australian Classification of Health Interventions; ICD-10, International Classification of Diseases, Tenth Revision.

MBS item number	AIHW ACHI code	ICD-10 diagnosis code
36806 - Ureteroscopy with extraction of stone from the ureter	36806-02 - Endoscopic extraction of ureteric calculus via ureteroscopy	N13.2 - Hydronephrosis with renal and ureteral calculous obstruction
36809 - Ureteroscopy with destruction of the stone in the ureter	36809-00 - Endoscopic fragmentation of ureteric calculus	N20.1 - Urolithiasis - calculus of the ureter
36549 - Ureterolithotomy	36803-02 - Endoscopic manipulation or extraction of ureteric calculus via ureteroscopy	N20.2 - Calculus of the kidney with calculus of the ureter
	36857-00 - Endoscopic manipulation or extraction of ureteric calculus without ureteroscopy	
	36549-00 - Ureterolithotomy	

Data handling 

Each admission was analyzed as an independent event, as national datasets do not contain unique patient identifiers and therefore cannot track readmissions, multiple procedures, or recurrence. Age data were grouped into standard categories (0-24, 25-44, 45-64, ≥65 years) for trend analysis.

Statistical analysis

Population-adjusted incidence rates were calculated using ABS annual population estimates. Temporal trends in diagnoses and interventions were assessed using univariable linear regression, with results reported as annual percentage change, coefficient of determination (*r*²), and *P*-values. A significance threshold of *P *< 0.05 was applied. All analyses were performed using GraphPad Prism (version 8.4.2, GraphPad Software, San Diego, CA). Confidence intervals were not applied because the analysis utilized complete national datasets rather than sampled data, and therefore, no sampling uncertainty was present.

## Results

Incidence of ureteric calculi and management trends

**Figure 1:**
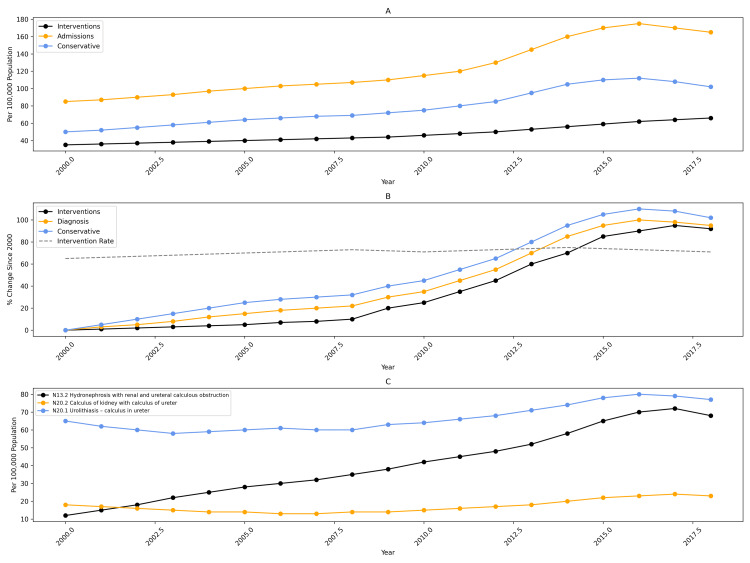
National trends in ureteric calculus diagnoses and management, 2000-2019. Population-adjusted trends in ureteric calculus diagnosis and management in Australia from 2000 to 2019. (A) Annual population-adjusted incidence of ureteric calculus-related admissions, interventions, and conservatively managed cases. (B) Relative population-adjusted percentage change in diagnoses, interventions, and conservative management, with the year 2000 set as the reference (0%). The intervention rate (percentage of diagnosed cases undergoing intervention) is also shown. (C) Population-adjusted incidence of ureteric calculus diagnoses was stratified by International Classification of Diseases, Tenth Revision (ICD-10) codes, including N13.2 (hydronephrosis with renal and ureteral calculous obstruction), N20.2 (calculus of kidney with calculus of ureter), and N20.1 (calculus of ureter).

In 2018-2019, there were 41,697 admissions for ureteric calculi in Australia, compared with 16,402 admissions in 2000-2001 - representing a 154% absolute increase. Population - adjusted incidence rose by 95%, increasing from 85.6 to 166.9 per 100,000 population (Figure 1A). Procedural interventions increased proportionally, rising from 6,519 to 16,166 procedures (147% increase), corresponding to a 90% increase in population-adjusted incidence (34.0 to 64.7 per 100,000). Conservatively managed admissions increased by 158% in absolute numbers and by 97% after adjusting for population (from 51.6 to 102.2 per 100,000).

Annual trend analysis demonstrated steady growth across all management pathways: ureteric calculus admissions increased by 3.65% per year (*r*² = 0.93, *P* < 0.01), interventions by 3.54% per year (*r*² = 0.89, *P* < 0.01), and conservative management by 5.23% per year (*r*² = 0.93, *P* < 0.01). Despite this growth, the national intervention rate remained stable, averaging 36.2% (range: 34.2%-39.8%) across the study period (Figure 1B). 

Across ICD-10 diagnostic categories, the largest increase occurred in hydronephrosis with calculus obstruction (N13.2), which rose from 11.4 to 66.2 per 100,000 population (481% increase; *r*² = 0.97, *P* < 0.01). Diagnoses of calculus of ureter (N20.1) increased by 20% (64.0 to 77.1 per 100,000; *r*² = 0.73, *P* < 0.01), while combined renal and ureteric calculi (N20.2) increased by 128% (10.3 to 23.5 per 100,000; *r*² = 0.87, *P* < 0.01) (Figure 1C).

Public vs. private sector interventions

**Figure 2:**
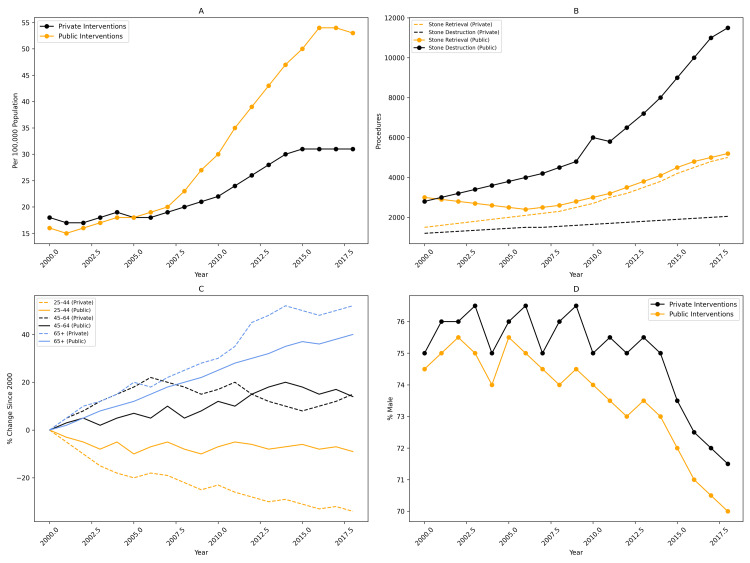
Public and private sector trends in ureteric calculus interventions in Australia, 2000-2019. Comparison of ureteric calculus interventions performed in Australian public and private hospitals between 2000 and 2019. (A) Population-adjusted intervention rates for ureteric calculi by sector. (B) Total number of interventions stratified by procedure type. (C) Percentage change in intervention rates by age group, with incidence in 2000 set as the reference (0%). (D) Proportion of male patients undergoing intervention for ureteric calculi.

Interventions increased steadily in both the public and private sectors; however, differences emerged over time. Between 2000-2001 and 2010-2011, intervention rates in the two sectors were similar. After 2010-2011, a divergence became evident, with public sector procedures increasing more rapidly. Between 2010-2011 and 2018-2019, public interventions grew by 7.2% per year compared with 4.5% per year in the private sector (Figure 2A). Endoscopic stone destruction procedures demonstrated particularly marked growth in the public sector, increasing by 323% since 2000-2001, compared with a 48% increase in the private sector (Figure 2B). In contrast, endoscopic stone retrieval procedures showed only modest growth, and ureterolithotomy remained rare, representing an average of 0.25% of all interventions. Overall, the proportion of procedures performed in public hospitals rose from 52% in 2000-2001 to 65% in 2018-2019, a 26% relative increase.

Age distribution of interventions

Substantial demographic shifts were observed across the study period. The most significant change was a rise in interventions among patients aged ≥65 years. In the private sector, this group increased from 22% to 34% of all interventions (53% relative increase), while in the public sector, the proportion rose from 25% to 28% (13% relative increase) (Figure 2C). In the private sector, interventions among individuals aged 45-64 years increased by 13%, whereas this age group declined by 8% in the public sector. The 25-44 age group remained the dominant demographic in private hospitals, although its proportion declined from 52% to 37%. In contrast, this age group remained stable in public hospitals (26%-25%)

Gender trends

**Figure 3:**
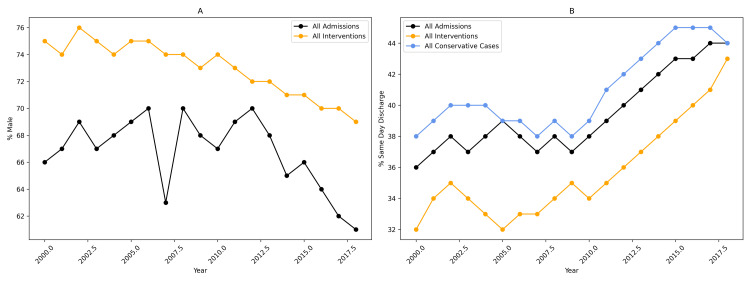
Sex distribution and same-day discharge trends in ureteric calculus admissions and interventions, 2000-2019. Temporal trends in patient sex distribution and same-day discharge rates for ureteric calculus–related care in Australia from 2000 to 2019. (A) Proportion of male patients among all ureteric calculus admissions and interventions over time. (B) Proportion of cases managed as same-day discharge, stratified by all admissions, all interventions, and conservatively managed cases.

The proportion of male patients admitted with ureteric calculi declined from 66% in 2000-2001 to 61% in 2018-2019, although this trend did not reach statistical significance (*r*² = 0.18, *P* = 0.075) (Figure 3A). Among those undergoing intervention, the proportion of males decreased from 75% to 70% (6% relative reduction; *r*² = 0.78, *P* < 0.01), corresponding to a 17% increase in female representation. Conservative management showed considerable year-to-year variability but remained nearly identical in 2000-2001 (55.22%) and 2018-2019 (55.24%).

Length of stay

Hospital length of stay decreased over time in both management pathways. Same-day discharge among conservatively managed patients increased from 37% in 2000-2001 to 45% in 2018-2019 (*r*² = 0.79, *P* < 0.01). A similar trend was observed in the interventional cohort, with same-day discharge rising from 31% to 44% (*r*² = 0.71, *P* < 0.01), achieving near equivalence with conservatively managed patients by 2018-2019 (Figure 3).

## Discussion

This national 19-year analysis demonstrates a substantial and sustained rise in ureteric calculus presentations in Australia, accompanied by proportional increases in procedural interventions. Despite the expanding burden of disease, the overall intervention rate remained stable at approximately one-third of admissions. This consistency suggests adherence to established evidence-based thresholds for surgical management, even as patient volumes increased [17]. 

In contrast to reports from the United Kingdom and the United States, where ureteroscopic intervention has risen disproportionately to disease incidence, the Australian trend shows procedural growth that closely mirrors the increase in presentations [10-12]. For example, in the United Kingdom, ureteroscopy increased by almost 50% over five years despite a minimal change in incidence [10]. These differing trends may be related to variations in healthcare systems and service delivery models, although these factors were not directly assessed in this study.

A notable observation in our study was the marked rise in diagnostic coding for hydronephrosis with calculus obstruction (N13.2), which increased more than fourfold over the study period. While this may imply a greater proportion of patients presenting with obstruction, it is more plausibly attributable to increased CT utilization, improved documentation, and evolving coding practices rather than a true shift in disease severity [10,11,15]. Hydronephrosis is a sign of obstruction rather than a complication, and the magnitude of the increase in this code should be interpreted cautiously in light of evolving coding practices [15].

The demographic trends observed also reflect global patterns in stone disease. Rising intervention rates among older adults are consistent with international observations of increasing urolithiasis prevalence in individuals aged over 65 years [1-3]. The gradual rise in interventions among women corresponds with national increases in obesity and metabolic syndrome - established risk factors for upper urinary tract calculi [18,19,20]. Obesity contributes to stone formation through multiple mechanisms, including elevated urinary excretion of calcium, oxalate, and uric acid, and reductions in urinary pH [20,21,22,23]. Although detailed metabolic data were not available in our dataset, these national trends likely contribute to the observed epidemiological shifts.

Same-day discharge rates for both conservative and interventional admissions increased steadily over the study period, reflecting broader improvements in perioperative care, anesthetic techniques, and postoperative analgesia. The safety and effectiveness of day case ureteroscopy have also been demonstrated internationally, with reported same-day discharge rates exceeding those seen in Australia [18]. Increasing adoption of ambulatory pathways likely reflects system-level efficiency initiatives and growing clinician confidence in streamlined care models.

Patterns of sector-specific utilization provide additional insight into the evolving landscape of endourological services in Australia. The disproportionate growth of public sector interventions, particularly endoscopic stone destruction, suggests expanding public access to flexible ureteroscopy and laser lithotripsy. Technologies that were historically more accessible in private hospitals [6-7]. This shift may reflect the centralization of acute stone services, declining private health insurance uptake among younger Australians, and increasing capability of public hospitals to manage complex or urgent stone presentations [6,7,22]. 

This study has several limitations inherent to administrative datasets. The absence of unique patient identifiers prevents assessment of recurrence, crossover between conservative and interventional pathways, or the cumulative burden of repeat procedures. Coding frameworks limit the availability of clinical detail, including stone size, location, composition, and imaging modality, and asymptomatic or incidental intrarenal stones are not reliably captured, resulting in underestimation of true nephrolithiasis prevalence [9,15]. Nonetheless, national datasets provide near-complete coverage of hospital-based symptomatic ureteric calculus presentations and offer robust insights into long-term epidemiological and procedural trends.

Overall, this study provides the most comprehensive national overview to date of ureteric calculus burden and management in Australia. The proportional rise in interventions, demographic shifts, and expanding public-sector role highlight growing resource demands associated with acute stone disease. Continued national surveillance supported by linked patient-level datasets will be essential to clarify the drivers of rising stone burden and to inform strategic planning, workforce distribution, and optimization of endourological services in the years ahead.

## Conclusions

Over the past two decades, Australia has seen a marked rise in ureteric stone incidence accompanied by increased endoscopic activity. Despite this expanding burden, national intervention rates have remained stable, reflecting consistent adherence to evidence-based thresholds for operative management. Demographic shifts, particularly higher intervention rates among older adults and the increasing representation of women, mirror broader changes in stone epidemiology. The growing reliance on public hospitals and the substantial rise in endoscopic procedures highlight evolving patterns of healthcare utilization and the maturation of endourological services.

To our knowledge, this study represents one of the longest national trend analyses of ureteric stone burden and management reported to date. These findings offer a definitive overview of Australia’s contemporary ureteric stone epidemiology and treatment patterns, underscoring the need for sustained investment in endourological capacity. Continued national surveillance will be essential to clarify the drivers of the rising stone burden and guide future service planning and workforce optimization.
